# Multicentre evaluation of the Boehringer Mannheim/Hitachi 911 Analysis System

**DOI:** 10.1155/S1463924693000264

**Published:** 1993

**Authors:** Z. Zaman, N. Blanckaert, Ch. Cobbaert, P. Gillery, P. Hagemann, H. Luthe, R. Motta, D. Patrono, M.-A. Jionsué, A. Torralba, M. J. Castiñeiras, X. Fuentes-Arderiu, W. Bablok, I. Domke, W. Stockmann

**Affiliations:** 1 Clinical Chemistry Laboratory University Hospitals Leuven Capucynenvoer 33–35 Leuven B 3000 Belgium; 2 Laboratoire Central de Biochimie Hôpital Robert Debré CHR Reims Rue Alexis Carrel Reims Cedex F 51092 France; 3 Zentrallaboratorium Kantonsspital Münsterlingen Münsterlingen CH 8596 Switzerland; 4 Abt. Klinische Chemie Zentrum Innere Medizin Georg-August-Universität Göttingen Robert-Koch-Str. 40 Göttingen D 37075 Germany; 5 Laboratorio Centralizzato Ospedale S. Orsola USL 28-B0 Nord Via Massarenti No. 9 Bologna I 40138 Italy; 6 Servei de Bioquímica Clínica Hospital Prínceps d'Espanya L'Hospitalet de Llobregat Barcelona E 08907 Spain; 7 Boehringer Mannheim GmbH Sandhofer Str. 116 Mannheim 31 D 68298 Germany

## Abstract

The analytical performance and practicability of the Boehringer Mannheim (BM)/Hitachi 911 analysis system have been assessed in a multicentre evaluation, which involved six laboratories from European countries. Analytes commonly used in classical clinical chemistry were tested in a core programme, which mainly followed the ECCLS guidelines. In addition, a satellite programme covered other analytes, such as proteins, drugs and urine analytes. In total, the study comprised more than 100 000 data items collected over a three-month period. The evaluation was supported with ‘Computer Aided Evaluation’ (CAEv) and telecommunications.

Acceptance criteria for the results were established at the beginning of the study. Nearly all of the analytes met the imprecision limits: within-run imprecision (as CVs) was 2% for enzyme and substrate assays, 1% for ISE methods and 5% for immunoassays; between-day imprecision was 3l% for enzyme and substrate assays, 2% for ISE methods and 10% for immunoassays.

No relevant drift effects (systematic deviation ≥ 3%) were observed over eight hours. The methods were linear over a wide range. Sample-related and reagent-dependent carry-over can be reduced to a negligible amount by integration of a softwarecontrolled wash-step.

Endogenous interferences were found for creatinine (Jaffé method) and uric acid assays (caused by bilirubin), for creatine kinase, creatine kinase MB isoform and γ-glutamyltransferase (caused by haemoglobin), and for immunoglobulin A (caused by lipaemia)

Accuracy was checked by an interlaboratory survey, recovery studies in control materials and method comparison studies. The survey showed that, with the exception of cholesterol and iron in two laboratories, the recovery of analytes did not deviate by more than 5%. Sixty-six of the 77 method comparisons performed met the acceptance criteria. The deviations of the remaining 11 results could be explained by differences in either calibration, application or by the use of different methods.

Practicability was assessed using a questionnaire which covered all of the important aspects of an analysis system in the clinical laboratory. Twelve groups of attributes out of 14 were rater higher for the BM/Hitachi 911 than for the present situation in the laboratories concerned. Especially high scores were given for the versatility group.

The acceptance criteria for the analytical performance of the BM/Hitachi 911 analysis system were fulfilled in all laboratory segments with few exceptions. The practicability exceeded the requirements in most of the attributes. The results of the study confirmed the usefulness of the system as a consolidated workstation in small- to medium-sized clinical laboratories and in STAT laboratories, or as an instrument for special analytes like proteins and drugs, or for urinalysis in large laboratories.


Journal of Automatic Chemistry, Vol. 15, No. 6 (November-December 1993), pp. 189-208

### Multicentre evaluation of the Boehringer Mannheim/Hitachi 911 Analysis System

**Z. Zaman, N. Blanckaert, Ch. Cobbaert**
*Clinical Chemistry Laboratory, University Hospitals Leuven, Capucynenvoer 33-35, B 3000 Leuven, Belgium*

**P. Gillery**
*Laboratoire Central de Biochimie, Hôpital Robert Debré CHR Reims, Rue Alexis Carrel, F 51092 Reims Cedex, France*

**P. Hagemann**

*Zentrallaboratorium, Kantonsspital Münsterlingen, CH 8596 Münsterlingen, Switzerland*

**H. Luthe**
*Abt. Klinische Chemie, Zentrum Innere Medizin, Georg-August-Universität Göttingen, Robert-Koch-Str. 40, D 37075 Göttingen, Germany*

**R. Motta, D. Patrono, M.-A. Jionsué**

*Laboratorio Centralizzato, Ospedale S. Orsola, USL 28-BO Nord, Via Massarenti No. 9, I 40138 Bologna, Italy*

**A. Torralba, M. J. Castiñeiras, X. Fuentes-Arderiu**
*Servei de Bioquímica Clínica, Hospital Prínceps d'Espanya, L'Hospitalet de Llobregat, E 08907 Barcelona, Spain*

**W. Bablok, I. Domke and W. Stockmann**
*Boehringer Mannheim GmbH, Sandhofer Str. 116, D 68298 Mannheim 31, Germany*

*The analytical performance and practicability of the Boehringer Mannheim (BM)/Hitachi 911 analysis system have been assessed in a multicentre evaluation, which involved six laboratories from European countries. Analytes commonly used in classical clinical chemistry were tested in a core programme, which mainly followed the ECCLS guidelines. In addition, a satellite programme covered other analytes, such as proteins, drugs and urine analytes. In total, the study comprised more than 100 000 data items collected over a three-month period. The evaluation was supported with 'Computer Aided Evaluation' (CAEv) and telecommunications.*

*Acceptance criteria for the results were established at the beginning of the study. Nearly all of the analytes met the imprecision limits: within-run imprecision (as CVs) was 2% for enzyme and substrate assays, 1% for ISE methods and 5% for immunoassays; between-day imprecision was 3% for enzyme and substrate assays, 2% for ISE methods and 10% for immunoassays.*

*No relevant drift effects (systematic deviation ≥3%) were observed over eight hours. The methods were linear over a wide range. Sample-related and reagent-dependent carry-over can be reduced to a negligible amount by integration of a software-controlled wash-step.*

*Endogenous interferences were found for creatinine (Jaffé method) and uric acid assays (caused by bilirubin), for creatine kinase, creatine kinase MB isoform and γ-glutamyltransferase (caused by haemoglobin), and for immunoglobulin A (caused by lipaemia).*

*Accuracy was checked by an interlaboratory survey, recovery studies in control materials and method comparison studies. The survey showed that, with the exception of cholesterol and iron in two laboratories, the recovery of analytes did not deviate by more than 5%. Sixty-six of the 77 method comparisons performed met the acceptance criteria. The deviations of the remaining 11 results could be explained by differences in either calibration, application or by the use of different methods.*

*Practicability was assessed using a questionnaire which covered all of the important aspects of an analysis system in the clinical laboratory. Twelve groups of attributes out of 14 were rater higher for the BM/Hitachi 911 than for the present situation in the laboratories concerned. Especially high scores were given for the versatility group.*

*The acceptance criteria for the analytical performance of the BM/Hitachi 911 analysis system were fulfilled in all laboratory segments with few exceptions. The practicability exceeded the requirements in most of the attributes. The results of the study confirmed the usefulness of the system as a consolidated workstation in small- to medium-sized clinical laboratories and in STAT laboratories, or as an instrument for special analytes like proteins and drugs, or for urinalysis in large laboratories.*

*Correspondence about this paper should be addressed to Dr Z. Zaman at the University Hospitals, Leuven.*

### Introduction

The Boehringer Mannheim (BM)/Hitachi 911 analysis system is the most recent medium-sized analysis system to be introduced to the market by Boehringer Mannheim GmbH. In addition to the well-accepted analytical performance and reliability of previous BM/Hitachi analysis systems, the new instrument has features making it attractive to different sections of clinical laboratories. These are in routine and emergency analysis (STAT), homogeneous immunoassays for the determination of proteins and drugs and urinalysis. Therefore the BM/Hitachi 911 has to be extremely flexible. This is achieved by incorporation of features such as automatic recognition of four different barcodes for sample identification, use of up to four reagents per test, variable reaction time, random analysis of serum and urine specimens using the same calibration curve, fully automated predilution of specimens and automatic calibration.

The versatility of the new instrument required the design of a comprehensive evaluation. Six European laboratories participated in the multicentre evaluation. Analytes of classical clinical chemistry were tested in a core programme, which mainly followed the ECCLS guidelines [1]. In addition, the behaviour of the system in different laboratory sections was tested in a satellite programme. The evaluators ran a much less extensive satellite evaluation in order to maintain an acceptable cost/benefit ratio. In total, the study included more than 100 000 data. Processing and analysis of the large data volumes was managed with a program system called 'Computer Aided Evaluation' (CAEv) and telecommunications.

CAEv integrates the definition of study protocols, performance of experiments in the laboratory, online/

0142-0453/93 $10.00 © 1993 Taylor & Francis Ltd.

offline data transmission and the immediate assessment and evaluation of the results [2]. The program runs on a standard PC under MS-DOS. It had previously been successfully applied to the multicentre evaluation of the BM/Hitachi 747 analysis system [3].

Telecommunications were used for the first time in an international multicentre evaluation. Installation of the necessary facilities in the participating laboratories allowed rapid and simple transfer of data to the centre coordinating the study.

The assessment of practicability was a further goal of the evaluation of the BM/Hitachi 911. The evaluators answered 200 questions each about the system. All results of the multicentre evaluation are presented in this paper.

### Description of the instrument

The BM/Hitachi 911 is a medium-sized selective access analyser with a capacity for 35 different tests, including three ion selective electrode (ISE) methods for sodium, potassium and chloride. The instrument specifications are listed in table 1. The throughput is 360 photometric tests/h; this is reduced by automatic sample predilution or additional wash steps which may be needed to eliminate reagent carry-over. The pipetting cycle for photometric tests, for the automatic predilution and for the wash steps, is 10 s and for the ISE methods 20 s.

Various measurements and calibration procedures that can be applied are:

(1) Endpoint measurements with/without sample blank within 16 min.

(2) Kinetic determinations with sample or substrate start (49 measuring points within 16 min).

(3) 'Fixed-time' measurements (two-point kinetic).

(4) Combination of kinetic and endpoint measurements with 'twin tests'.

(5) Potentiometric measurements with the ISE units within 20 s at 37°C.

(6) Serum indices for the characterization of haemolytic, icteric and lipaemic sera.

(7) Prozone check (detection of antigen excess).

(8) Linear and four nonlinear modes of calibration, the frequency of which is controlled by the instrument (auto-calibration).

*Table 1. BM/Hitachi 911 instrument specifications.*

| | | |
|---|---|---|
| 1 | Type of instrument | Discrete selective multianalyser. |
| 2 | Test channels | 32, with ISE-module 35. |
| 3 | Test procedures | Endpoint, endpoint with sample blank, kinetic with serum or substrate start, fixed-time kinetic, combination of two endpoint tests, endpoint and kinetic tests, two kinetics tests performing two tests in one cuvette, two prozone check procedures, measurement with ISE, linear calibration and four nonlinear modes of calibration, autocalibration, two-point recalibration, isoenzyme calibration, serum indices indicating haemolytic, icteric and lipaemic specimens. |
| 4 | Throughput | Maximum 360 photometric tests/h, with ISE-module 720 tests/h. |
| 5 | Sampling system | Turntable with 115 positions in total, arranged in three concentric rings:<br>—50 positions in the outer ring for routine samples<br>—40 positions in the middle ring<br>— 3 for wash solutions<br>—20 for STAT samples<br>—17 for standards/calibrators<br>—25 cooled positions in the inner ring for 17 standard and 8 control samples.<br>Primary tubes from 13 to 16 mm diameter and 75 to 100 mm length, secondary sample cups with 2 ml and microcups with 0·5 ml maximum volume. Barcode identification of primary tubes. Codes: code bar NW 7, code 32, 2 out of 5 interleaved, code 128. Different codes and different tube sizes can be used within one sample disk. |
| 6 | Sample pipettor | 3 to 50 µl (in steps of 1 µl), imprecision $<1\%$; for ISE same pipettor 15 µl for the three determinations of Na, K, Cl. |
| 7 | Reagent cooling | Cold water circuit, refrigerator temperature 5 to 15°C. |
| 8 | Reagent bottles | 20, 50 and 100 ml sizes, 2 reagent disks with 33 bottle positions, one position reserved for cuvettes rinsing solution. Disk 1 for reagent 1 and 4, disk 2 for reagent 2 and 3. |
| 9 | Reagent dispenser | 2 reagent pipettors for dispensing reagents 1 to 4, 50 to 350 µl (in steps of 1 µl). |
| 10 | Mixing procedure | 2 stirrers mix the reaction solution independently after addition of each reagent. |
| 11 | Reaction rotor | Turntable with 120 cuvettes; half rotation (60 cuvettes + 1) in 10 s = one working cycle. |
| 12 | Reaction cuvettes | Special plastic cuvettes, semidisposable.<br>Volume required: minimum 250 µl, maximum 500 µl.<br>Optical path length: 6 mm. |
| 13 | Reaction cycle | 3, 4, 5, 10 and 16 minutes reaction time corresponding to 10, 13, 15, 31 and 49 measuring points. Average time between two measuring points: 20 s. |
| 14 | Temperature control | Water-bath, 37° ± 0·1°C. |

*Table 1 (continued).*

| | | |
|---|---|---|
| 15 | Photometer | —Single beam photometer with 12 available wavelengths: 340, 376, 415, 450, 480, 505, 546, 570, 600, 660, 700 and 800 nm; mono- or bichromatic measurement with free selectable wavelengths combination.<br>—Light source: halogen lamp. Detector- silicone photodiode.<br>—Wavelength adjustment: fixed, via a grating chromator, inaccuracy at 340 nm ± 2 nm and at 405 to 800 nm ± 5 nm.<br>—Half band width: 4 nm in the UV range<br>10 nm in the visible range<br>—Photometric range of linearity: A = 0 – 2·5 at 340 nm<br>—Photometric resolution: A = 0·0002<br>—Photometric inaccuracy: max. 1% at 2·0 abs. |
| 16 | Ion-selective electrodes | —Indirect potentiometry; flow-through electrode with liquid membrane.<br>—Reference electrode: liquid membrane<br>—Dilution ratio: 1:31<br>—Incubation temperature: 37 ± 0·1°C<br>—Calibration: 2-point with compensation<br>—Measuring cycle: 20 s<br>—Measuring range in serum / in urine<br>$Na^+$ 80–180 / 10–250 mmol/l<br>$K^+$ 1·5–10 / 1–100 mmol/l<br>$Cl^-$ 60–120 / 10–250 mmol/l<br>—Consumption of ISE solution per sample:<br>diluent: 450 µl<br>internal standard: 1050 µl<br>KCl solution 130 µl |
| 17 | Data processing | —Data input: via alpha numeric keyboard and item select keys.<br>—Data control: CRT 14 inches colour monitor.<br>—Data output: matrix printer, 80 characters per line, 220 characters per second.<br>—2 floppy disk drives, 3·5 inches, 1 system disk and 1 data disk for storage of 800 routine samples and 200 STAT samples.<br>—Interface: RS 232 C. Bidirectional link with a host computer. |
| 18 | Water supply | —Internal reservoir with an external supply.<br>Quality: ≤ 1 µS<br>Consumption during operation: 30 l/h |
| 19 | Ambient temperature | 15 to 32°C |
| 20 | Relative humidity | 45 to 85% |
| 21 | Physical dimensions | Analyser unit / Operator unit<br>Width 1·03 m / 0·48 m<br>Depth 0·76 m / 0·65 m<br>Height 1·17 m / 1·25 m |
| 22 | Weight | Approx. 350 kg |

The manufacturer provides an application disk to load and store the application settings for the barcoded system reagents. These can be inserted in any free position of the reagent disk. For the use of non-barcoded reagents, the operator must define the appropriate application settings.

Specimens (3–50 µl volume per test) are processed either from primary tubes (5–10 ml), secondary cups (2 ml) or microcups (500 µl). The primary tubes can be identified by four different types of barcodes. The RS 232 interface allows a bidirectional communication to a host computer.

One hundred and twenty plastic cuvettes (d = 6 mm) are arranged on a rotor which rotates in a water bath at 37°C (±0·1°C). The cuvettes pass through the beam of the photometer every 20 s (12 fixed wavelengths between 340 and 800 nm, mono- or bichromatic). The photometric range of linearity at 340 nm extends to an absorbance of 2.5.

Two pipettors (50–350 µl) transfer the reagent into a cuvette. The average reagent consumption is 300 µl per determination.

Up to four reagents can be pipetted per test. For several STAT tests the second reagent is added 1·3 min after the sample pipetting and for routine tests after 5 min. If necessary, a fourth reagent can be pipetted after 10 min. The results of eight STAT tests are available within 6 min.

### Materials and methods

### *Instruments and reagents*

The methods and instruments used in the study are listed in table 2. The same lot of reagents was used in all evaluation centres for each method. Reagents for the BM/Hitachi instrument were available in system packs containing barcoded bottles.

*Table 2. Analytes, methods and comparison instruments.*

| Analyte | Abbreviation | Method on BM/Hitachi 911 | Comparison instruments | Comparison methods |
|---|---|---|---|---|
| Pancreatic α-amylase | PAMYL | Ethyliden-$G_7$ PNP as substrate (EPS) | BM/Hitachi 704, 747 | Same as BM/Hitachi 911 |
| Aspartate aminotransferase | ASAT | According to IFCC and standard optimized | BM/Hitachi 704, 747 | Same as BM/Hitachi 911 |
| Creatine kinase | CK | Standard optimized | BM/Hitachi 717, 737, 747 | Same as BM/Hitachi 911 |
| Creatine kinase MB isoform | CK-MB | Standard optimized, immunoinhibition | BM/Hitachi 717, 747 | Same as BM/Hitachi 911 |
| γ-Glutamyltransferase | GGT | L-γ-glutamyl-3-carboxy-4-nitroanilide as substrate | BM/Hitachi 717, 737, 747 | Same as BM/Hitachi 911 |
| β-N-Acetylglucosaminidase | BNAG | Chlorophenol red (CPR)-NAG as substrate | COBAS® Fara (Hoffmann-LaRoche) | Same as BM/Hitachi 911 |
| Calcium | CA | o-Cresolphthalein complexone | BM/Hitachi 747, Atomic absorbance spectrometer Perkin Elmer 1100 B | Same as BM/Hitachi 911 Atomic absorption spectroscopy |
| Cholesterol | CHOL | Cholesterol oxidase/ p-aminophenozone | BM/Hitachi 704, 747 | Same as BM/Hitachi 911 |
| Creatinine | CREA | Jaffé (kinetic) without deproteinization | BM/Hitachi 704, 717, 747, CX3 (Beckman) | Same as BM/Hitachi 911 |
| Iron | FE | FerroZine without deproteinization | BM/Hitachi 704, 747, COBAS® Fara (Hoffmann-LaRoche) | Same as BM/Hitachi 911 |
| Fructosamine | FRUCT | Reduction of nitroblue tetrazolium chloride | Isamat® | Same as BM/Hitachi 911 |
| Total protein | TP | Biuret | BM/Hitachi 704, 717, 747 | Same as BM/Hitachi 911 |
| Uric acid | UA | Enzymatic PAP | BM/Hitachi 704, 717, 747 | Same as BM/Hitachi 911 |
| C-Reactive protein | CRP | Turbidimetry | Behring Nephelometer | Fixed-time nephelometry |
| Ferritin | FT | Turbidimetry | Stratus® II analyser | Fluorescence immunoassay |
| Immunoglobulin A | IGA | Turbidimetry | Behring Nephelometer | Fixed-time nephelometry |
| Transferrin | TF | Turbidimetry | ARRAY® analyser (Beckman) | Rate nephelometry |
| Albumin in urine | MAU | Turbidimetry | COBAS® Fara (Hoffmann-LaRoche), ARRAY® analyser (Beckman) Manual | Turbidimedtry Rate nephelometry Radioimmunoassay |
| Phenobarbital | PHEBA | Homogeneous immunoassay, CEDIA® Phenobarbital | Abbott TDx | Fluorescence polarization immunoassay |
| Phenotoin | PHENY | Homogeneous immunoassay, CEDIA® Phenytoin | Abbott TDx | Fluorescence polarization immunoassay |
| Theophylline | THEO | Homogeneous immunoassay, CEDIA® Theophylline | Abbott TDx | Fluorescence polarization immunoassay |
| Digoxin | DIG | Homogeneous immunoassay, CEDIA® Digoxin | Abbott TDx | Fluorescence polarization immunoassay |
| Sodium | NA | ISE | BM/Hitachi 747, CX3 (Beckman), Flame spectrometer SCM 6341 (Eppendorf) | ISE Flame atomic emission spectrometry |
| Potassium | K | ISE | BM/Hitachi 747, CX3 (Beckman), Flame spectrometer SCM 6341 (Eppendorf) | ISE Flame atomic emission spectrometry |
| Chloride | CL | ISE | BM/Hitachi 747, CX3 (Beckman), Chloride analyser 925 (Corning) | ISE Coulometry |

*Control materials*

Imprecision and quality control studies were performed with lyophilized control sera (Boehringer Mannheim GmbH) and control urines (Boehringer Mannheim GmbH, Bio-Rad). Detailed information is shown in the appendix (table 10).

*Calibration*

During the familiarization period, a fixed-factor was determined for the enzyme tests in three independent calibration runs per day on three consecutive days. The same lot of the calibrator for automated systems (Boehringer Mannheim GmbH) was used for this purpose. The mean of the nine calibration runs was then taken as the factor, provided that the range of all results did not exceed 3% of the mean.

During experiments for the between-day imprecision and method comparison (21 working days) autocalibration of the analytical system was tested. The autocalibration is triggered by an analyte-dependent calibration interval. In order to avoid any additional effects on the results, a 'start-up' calibration was activated before the remaining experiments. Detailed information about the calibrators employed is shown in the appendix (table 10).

*Evaluation protocol*

The versatility of the BM/Hitachi 911 analysis system was tested in a core programme and a satellite programme. The core program had been used for evaluations of the BM/Hitachi 704, 717 and 747 analysis systems [3–5].

The 13 analytes tested in the core programme were pancreatic α-amylase, aspartate aminotransferase, creatine kinase, γ-glutamyltransferase, calcium, cholesterol, creatinine, iron, total protein, uric acid, sodium, potassium, and chloride. This programme included a familiarization period, an initial trial and a main trial. The protocol of the main trial is shown in table 3. The main trial was split between two groups, each consisting of three laboratories, which tested the same set of analytes. For the studies of linearity, drift and sample-related carry-over, the different methods were divided between the six evaluators.

The satellite programme covered other analytes such as proteins (C-reactive protein, ferritin, immunoglobulin A, transferrin), drugs (phenobarbital, phenytoin, theophylline, digoxin) and urinalysis (albumin, β-N-acetylglucosaminidase, creatinine, sodium, potassium, chloride). In addition, creatinine kinase MB isoform and fruc-

*Table 3. Protocol of the multicentre evaluation (core programme, main trial)*.*

| Imprecision |
|---|
| Within-run<br>On three different days, each day one run with 21 aliquots<br>—Three control materials with different concentrations of the analyte.<br>—One human serum pool at the decision level.<br>Between-day<br>—Three control materials with different concentrations of the analyte over 21 days (evaluation and comparison instrument). |
| **Drift** |
| —Two control sera and the calibrator were analysed every 30 min over 8 h to test eight methods (aspartate aminotransferase, creatine kinase, γ-glutamyltransferase, calcium, iron, creatinine, total protein and uric acid).<br>—At zero hour, triplicate measurements were performed and the median was taken as the base value.<br>—The percentage recovery of the base value was taken for assessing drift effects. |
| **Analytical range limits [7]** |
| —A high level sample was diluted with a low level sample to obtain a series of eleven concentrations with two being the baseline samples and nine intermediate concentrations.<br>—Triplicate measurements on the 11 concentration levels were performed and the median for each level was calculated.<br>—The regression line (Passing/Bablok regression) was calculated using a range covering five concentration levels which was assumed to be linear.<br>—The target values for all concentration levels were calculated from the regression lines. |
| **Carry-over** |
| Sample-related<br>Model of Broughton [11]:<br>—Measurements of five aliquots of a high-concentration sample ($h_1 \cdots h_5$) are followed by<br>—Measurements of five aliquots of a low-concentration sample ($l_1 \cdots l_5$). The experiment is repeated 10 times.<br>If a carry-over effect exists, $l_1$ is the most influenced, $l_5$ the least influenced aliquot.<br>Reagent-dependent:<br>One control material was used.<br>Assay A influences assay B. |

* For details, see reference [3].

(*continued*)

*Table 3 (continued).*

—Carry-over caused by the cuvettes
In a first step, reagents for assay A were requested 21 times. Just after the dispensing of the reagents for the 21st aliquot, the analyser was stopped. In a second step, reagents for assay B were requested 42 times. The first 21 determinations were performed in the cuvettes which previously had contained the reagents for assay A. These determinations would show carry-over effects whereas the last 21 determinations would be uninfluenced. The difference of the medians of both series was the carry-over.
—Carry-over caused by reagent probes and stirrers
Assay B was carried out 21 times. In the second step test A and B were alternately performed 21 times. The carry-over was the difference between the medians of both series.

Interference

Protocol of Glick [8]
A specimen with concentrations at the decision level was spiked with the interfering substance and 10 serial dilutions were prepared with the same baseline specimen. The different analytes were measured in triplicate. The percentage recovery of the baseline value for each concentration level was calculated.

Accuracy

Calibration
The calibrators of BM/Hitachi 911 and of the comparison instrument were both run on each instrument.

Quality control in three control materials
—Median, calculated from the second of duplicate measurements over 21 days.

Interlaboratory survey
—One control material with concentrations not known to the evaluators.
—Median, calculated from the second of duplicate measurements over 10 days.

Method comparison in fresh human specimens
—10 to 15 specimens were analysed each day for 10 days on the BM/Hitachi 911 and on the comparison instruments. The total number of specimens covered the entire analytical range.
—Comparison of the methods by calculation of the Passing/Bablok regression line [10].

In the satellite programme within-run imprecision was determined using one or two control materials and one human pool. Between-day imprecision was studied over 10 days with one or two control materials.
Drift, linearity, carry-over and interference studies were carried out only for selected analytes.

tosamine assays were run in this part of the evaluation study. The results of these analytes are presented together with those of the core programme.

The protocol included quality specifications which were agreed at the evaorators' first meeting: these are described later in this paper.

### *Assessment of practicability*

Practicability was assessed with the aid of a recently published questionnaire [6] comprising about 200 questions which covered all important aspects of an analysis system in the clinical laboratory. The questions were summarized into 14 groups, as shown in table 4. They were related to the installation of the analyser, organization of work, quality assurance and miscellaneous characteristics. A first version of the questionnaire had already been used for the assessment of practicability of BM/Hitachi 747 [3].

Grading was in comparison with the evaluators' present laboratory situation. The assessment was based on a scale from 0 to 10: a score of 0 meant unimportant, useless or poor, and a score of 10 absolutely necessary or excellent. A score of 5 could be interpreted as being acceptable or comparable with the present laboratory situation. The grading was divided into three classes. Scores of up to 3·3 meant 'did not meet the requirements', scores from 3·4 to 6·7 'meets the requirements', and scores from 6·8 to 10 'exceeded the requirements'.

### Results

### *Imprecision*

Acceptance criteria for imprecision were based on statistical error propagation [3]. For within-run imprecision in participating laboratories the median of the CVs should not exceed 2% for classical clinical chemistry analytes (enzymes and substrates) in serum (only in the tables is a differentiation made between serum and plasma) and urine at all concentrations tested. The accepted CV was reduced to 1% for the determination of electrolytes by ISE. Taking into consideration the problems associated with immunoassays, such as nonlinear calibration or analytical sensitivity, it was agreed that CVs of less than 5% would be acceptable for these assays.

Within-run imprecision data based on results from all the control sera from all laboratories are shown in figures 1–3, and data for individual control sera are given in the appendix (table 11). The medians of all analytes met the acceptance criteria, except for phenytoin and digoxin in the low level control. In individual control sera, results exceeding the acceptance limits were obtained for

*Table 4. Assessment of practicability—groups of attributes.*

| | Number of items | Contents |
|---|---|---|
| Installation | | |
| (1) Environment | 10 | Installation conditions (space, power and water supply, waste, noise) |
| (2) Spatial arrangements | 14 | Location of instrument components |
| (3) Training/operation | 14 | Operation (demand on skills)<br>Training course<br>Instruction manual |
| Organization of work | | |
| (4) Start-up/shut-down | 5 | Duration<br>Availability |
| (5) Sample processing | 31 | Tray<br>Loading<br>Vessels<br>Predilution<br>Volumes<br>Volume detection<br>Identification |
| (6) Reagent handling | 17 | Preparation<br>Loading<br>Identification<br>Stability<br>Exchange<br>Volume |
| (7) Work flow | 23 | Available tests<br>Test request<br>STAT procedure<br>Rerun<br>Result presentation |
| (8) Timing | 7 | Throughput<br>Startup time<br>STAT time<br>Calibration<br>Operator time |
| Quality assurance | | |
| (9) Monitoring | 5 | Sample processing<br>Reagent processing<br>Measuring process<br>Temperature control<br>Photometer control |
| (10) Calibration | 23 | Materials<br>Monitoring<br>Procedure<br>Frequency |
| (11) Quality control | 12 | Number of controls<br>Statistics<br>Real time/daily/cumulative<br>Alarms<br>Presentation |
| Miscellaneous | | |
| (12) Data processing environment | 15 | Hardware<br>Software<br>Communication |
| (13) Versatility | 3 | Analysis modes<br>Ease of applications |
| (14) Maintenance/troubleshooting | 15 | Duration and frequency<br>Combinations of maintenance functions<br>Volumes for priming<br>Trouble-shooting<br>Alarms<br>Availability of technical service<br>Power break-down |

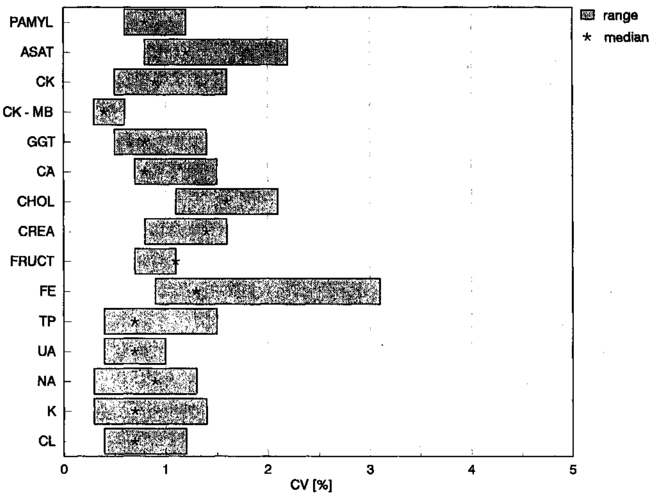

*Figure 1. Within-run imprecision for classical clinical chemistry analytes and electrolytes, based on control material data from all laboratories in the study.*

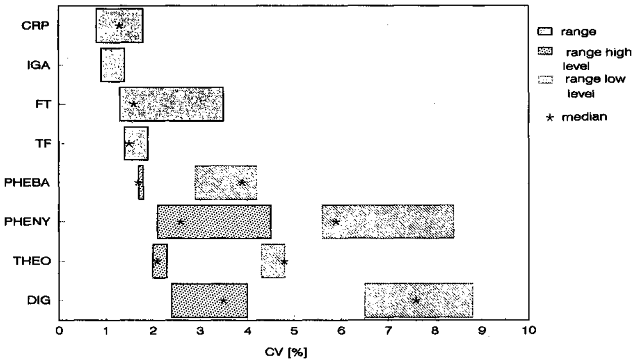

*Figure 2. Within-run imprecision for homogeneous immunoassays, based on control material data from all laboratories.*

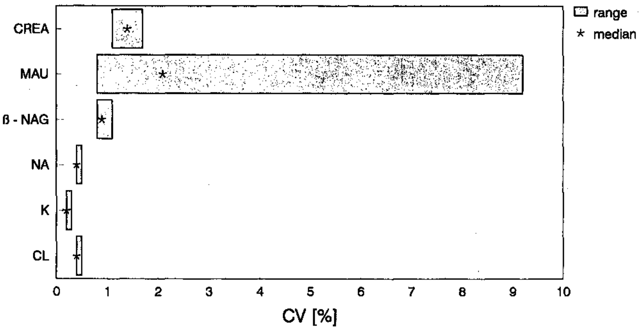

*Figure 3. Within-run imprecision for analytes in urine, based on control material data from all laboratories.*

cholesterol, iron, sodium, potassium, chloride and for albumin in urine.

All analytes which had not met the quality specifications in control materials showed acceptable CVs in human materials (table 5). Only the CV of creatine kinase MB isoform exceeded the acceptance criteria.

Between-day imprecision was defined as acceptable if median CVs for electrolytes were $\leq 2\%$, for homogeneous immunoassays $\leq 10\%$ and for the remaining analytes investigated in serum and urine $\leq 3\%$.

*Table 5. Within-run imprecision in a normal human serum, plasma or urine pool ($N = 21$). The results of the pools with concentrations close to the decision level are shown.*

| Analyte | Unit | Mean | CV % |
|---|---|---|---|
| Pancreatic α-amylase | U/l | 71 | 1·7 |
| Aspartate aminotransferase | U/l | 30 | 1·3 |
| Creatine kinase | U/l | 149 | 0·8 |
| Creatine kinase, MB isoform | U/l | 10 | 3·1 |
| γ-Glutamyltransferase | U/l | 46 | 0·9 |
| Calcium | mmol/l | 2·2 | 0·9 |
| Cholesterol | mmol/l | 5·1 | 1·7 |
| Creatinine | µmol/l | 99 | 1·6 |
| Fructosamine | µmol/l | 250 | 1·4 |
| Iron | µmol/l | 13 | 1·8 |
| Total protein | g/l | 70 | 0·4 |
| Uric acid | µmol/l | 350 | 1·0 |
| Sodium | mmol/l | 141 | 0·5 |
| Potassium | mmol/l | 4·0 | 0·6 |
| Chloride | mmol/l | 101 | 0·6 |
| C-Reactive protein | mg/l | 14 | 3·2 |
| Immunoglobulin A | g/l | 1·8 | 2·5 |
| Ferritin | µg/l | 239 | 2·2 |
| Transferrin | g/l | 2·6 | 2·0 |
| Phenobarbital | µg/ml | 16 | 2·8 |
| Phenytoin | µg/ml | 8·6 | 2·9 |
| Theophylline | µg/ml | 12 | 3·0 |
| Digoxin | ng/ml | 1·9 | 4·0 |
| β-N-Acetylglucosaminidase* | U/l | 10 | 0·6 |
| Creatinine* | mmol/l | 1·0 | 1·0 |
| Albumin* | mg/l | 33 | 1·4 |
| Sodium* | mmol/l | 110 | 0·4 |
| Potassium* | mmol/l | 35 | 0·5 |
| Chloride* | mmol/l | 96 | 0·5 |

*Analytes in urine.

The medians and ranges of CVs in control materials analysed on BM/Hitachi 911 are shown in figures 4–6; results obtained in individual control sera are presented in the appendix (table 11). The medians met the defined quality specifications in all cases. Individual CVs exceeding the acceptance criteria were obtained for calcium, cholesterol, creatinine, sodium, chloride, phenytoin, digoxin in serum and for albumin in urine. The results of the comparison instruments were of the same order as those of BM/Hitachi 911 (data not shown).

### *Analytical range limits*

It was desirable for the measuring range to cover the greatest part of the physiological and pathophysiological range. Analytical range limits were established by determining the difference between the measured values and the target values from the dilution experiments as described by Bablok [7]. In the upper range, a method was defined to be linear if this difference was less than 5%. The lower range was judged on the basis of such pragmatic considerations as absolute differences between measured and target values, and the diagnostic relevance of these differences. In the case of multi-point calibration, a method could be called linear if a change in the target concentration led to a proportional change in the measured concentration.

A wide linearity range was obtained for all clinical chemistry analytes in serum (table 6). The lower limit tested for creatinine and calcium was found to be 30 µmol/l, and 0·3 mmol/l, respectively.

In urine, the required high linearity ranges were reached for all analytes. Linearity in the low range was analysed for albumin and sodium only. The albumin method was found to be nonlinear below 25 mg/l and the sodium determination below 10 mmol/l.

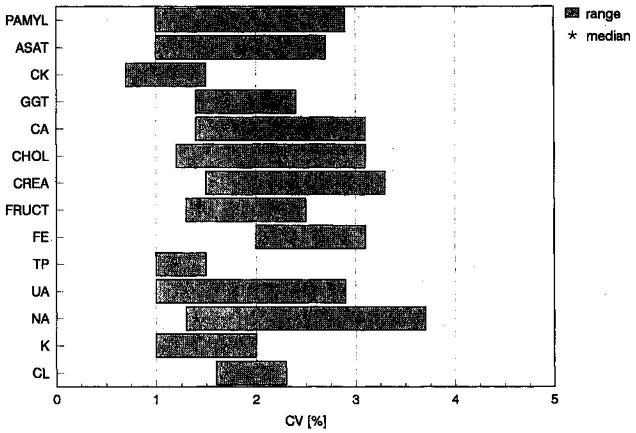

*Figure 4. Between-day imprecision for classical clinical chemistry analytes and electrolytes, based on control material data from all laboratories.*

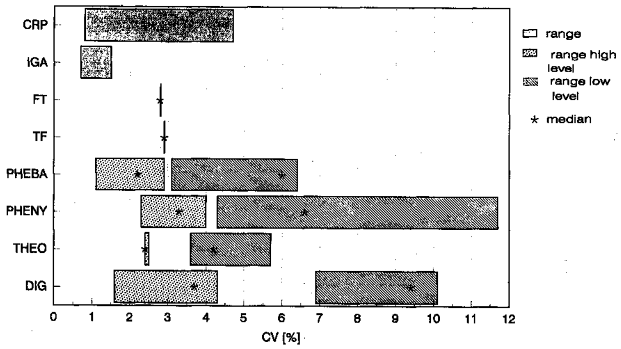

*Figure 5. Between-day imprecision for homogeneous immunoassays, based on control material data from all laboratories.*

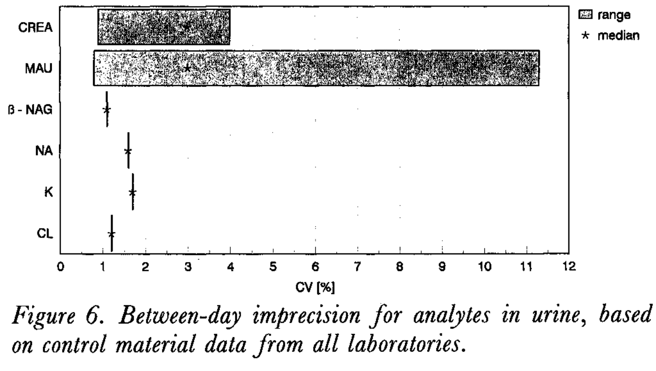

*Figure 6. Between-day imprecision for analytes in urine, based on control material data from all laboratories.*

*Table 6. Analytical ranges on the BM/Hitachi 911.*

| Analyte | Sample material | Unit | Range: Tested between | Range: Found between |
|---|---|---|---|---|
| Aspartate aminotransferase (IFCC method) | serum | U/l | 14 – 870 | 14 – 870 |
| Creatine kinase | serum | U/l | 10 –1520 | 10 –1520 |
| γ-Glutamyltransferase | serum | U/l | 10 –1250 | 10 –1250 |
| Pancreatic α-amylase | serum | U/l | 16 –2400 | 16 –2400 |
| Calcium | serum | mmol/l | 0 – 6 | 0·3– 6 |
| Cholesterol | plasma | mmol/l | 2·9– 21·5 | 2·9– 21·5 |
| Creatinine | serum | μmol/l | 0 –2000 | 30 –2000 |
| Iron | serum | μmol/l | 0·9– 20 | 0·9– 20 |
| | | | 10 – 215 | 10 – 215 |
| Total protein | plasma | g/l | 8 – 175 | 8 – 175 |
| Uric acid | plasma | μmol/l | 0 –1200 | 0 –1200 |
| C-Reactive protein* | serum | mg/l | 0 – 250 | 0 – 250 |
| Immunoglobulin A | serum | g/l | 1 – 6·3 | 1 – 6·0 |
| β-N-Acetylglucosaminidase | urine | U/l | 0 – 78 | 0 – 78 |
| Creatinine | urine | mmol/l | 1·5– 23 | 1·5– 23 |
| Albumin | urine | mg/l | 0 – 500 | 20 – 500 |
| Sodium | urine | mmol/l | 0 – 200 | 10 – 200 |
| Potassium | urine | mmol/l | 0 – 115 | 0 – 115 |
| Chloride | urine | mmol/l | 0 – 215 | 30 – 215 |

*Multi-point calibration, see discussion in text.

*Drift*

Drift effects were not accepted if a systematic deviation from the initial value exceeded 3%. Over an eight-hour-period, no drift effects were observed in any of the 10 methods tested.

*Calibration stability*

Calibration stability based on individual calibration frequency claims was observed over a period of 21 working days. No deviations in recovery exceeding 3% were found.

*Carry-over*

Carry-over effects were assessed on the basis of the observed change in recovery of an analyte. Instead of adapting an individual deviation for each analyte it was decided to use the within-run imprecision system performance. This was defined as a change of less than twice the standard deviation being acceptable.

Sample-related carry-over was only tested for analytes with a large physiological range and between urine and serum specimens. For the potassium assay, a slight carry-over effect (0·17 mmol/l) was observed from urine to serum with a concentration ratio of 77:1 (table 7). This was rated as being of no clinical relevance.

Previous experience with BM/Hitachi systems had revealed reagent dependent carry-over caused by the cuvettes for the combination triglycerides/lipase. This effect was avoided by activating a special wash solution step within the normal cleaning procedure for the cuvettes on BM/Hitachi 911.

Carry-over caused by the reagent probes and the stirrers was tested for the combinations triglycerides/lipase and aspartate aminotransferase/lactate dehydrogenase. This resulted in an elevation of lipase activities by about 400 U/l and of lactate dehydrogenase activities by about 30 U/l. Activation of a software-controlled wash-step for these combinations reduced the carry-over effects to a negligible amount (< 10 U/l).

*Interferences*

According to Glick *et al.* [8], a method is resistant to interferences if the deviation between the baseline value and the measured value is less than 10%. Only methods not fulfilling this acceptance criterion are shown in the interferograms of bilirubinaemia, lipaemia and haemolysis (figures 7–9).

Of the 13 methods tested only two showed interference by bilirubin. Decreased values were found for the creatinine (Jaffé method) and for the uric acid assays at

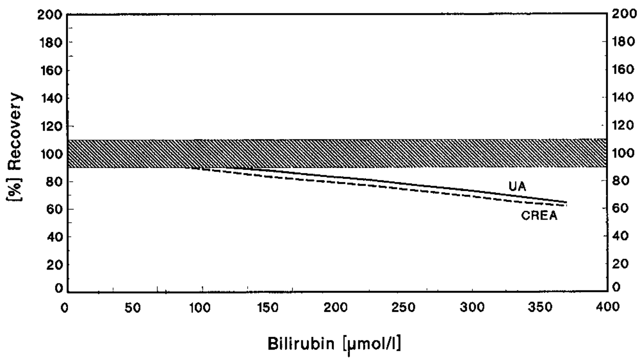

*Figure 7. Interference of bilirubin. The hatched zone represents 90 to 110% recovery of the baseline value. Analytes tested were: creatine kinase, γ-glutamyltransferase, creatinine, total protein, uric acid, sodium, potassium, chloride, C-reactive protein, immunoglobulin A, theophylline, phenobarbital, and phenytoin.*

*Table 7. Sample-related carry-over.*

| Analyte (sample materials) | Unit | Median of high concentration | Median of low concentration | Ratio h: l | Median of $l_1 - l_5$ | Standard deviation $l_3, l_4, l_5$ |
|---|---|---|---|---|---|---|
| Creatine kinase (serum/serum) | U/l | 6282 | 12·0 | 524 | 0·30 | 0·42 |
| Ferritin (serum/serum) | μg/l | 5480 | 196 | 28 | 0·80 | 12·8 |
| Creatinine (urine/plasma) | μmol/l | 35100 | 58·1 | 604 | 0·40 | 1·25 |
| Albumin (serum/urine) | mg/l | 54300 | 16·5 | 3290 | 0·60 | 0·28 |
| Albumin (plasma/urine) | mg/l | 39400 | 21·3 | 1850 | −0·05 | 0·21 |
| Potassium (urine/serum) | mmol/l | 224 | 2·9 | 77 | 0·17 | 0·02 |
| Potassium (urine/plasma) | mmol/l | 161 | 4·0 | 40 | 0·04 | 0·02 |

h, l specimen of high, low concentration.
$l_1$ specimen of low concentration, influenced by h.
$l_3, l_4, l_5$ specimen of low concentration, uninfluenced.

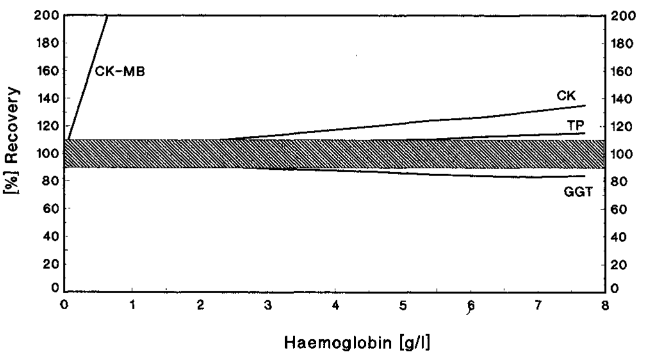

*Figure 8. Interference of haemolysis. The hatched zone represents 90 to 110% recovery of the baseline value. Analytes tested were: creatine kinase, creatine kinase MB isoform, γ-glutamyltransferase, creatinine, total protein, uric acid, and fructosamine.*

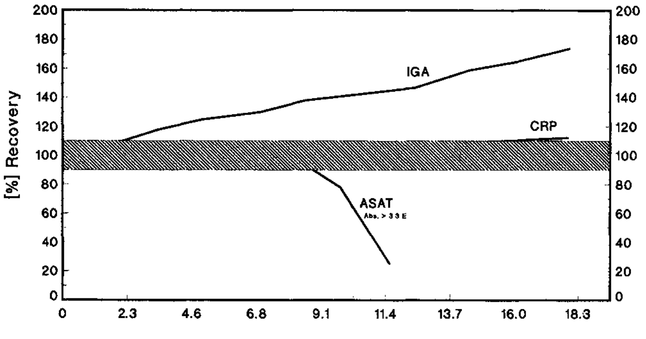

*Figure 9. Interference of lipaemia. The hatched zone represents 90 to 110% recovery of the baseline value. Analytes tested: pancreatic amylase, aspartate aminotransferase, calcium, cholesterol, iron, sodium, potassium, chloride, C-reactive protein, immunoglobulin A, transferrin, ferritin, phenobarbital, phenytoin, theophylline, and digoxin.*

bilirubin concentrations above 120 μmol/l and 140 μmol/l, respectively (figure 7). Similar effects have been observed on other BM/Hitachi instruments [3].

Haemolysis caused interference with four of the methods tested (figure 8). Activities of creatine kinase and creatine kinase MB isoform were increased while that of γ-glutamyltransferase decreased. The protein assay gave positive bias with haemolysis at high haemoglobin concentrations (>4 g/l).

Two of the 16 methods evaluated were susceptible to interference by lipaemia (figure 9). While immunoglobulin A concentration was increased, an opposite effect was observed on the activities of aspartate aminotransferase. A slight increase was also observed in concentrations of C-reactive protein (+12%) at high concentrations of triglycerides (>1500 mmol/l).

### *Accuracy*

#### *Interlaboratory survey and quality control*

Five of the six laboratories participated in an interlaboratory survey in which the concentrations of 15 analytes were determined on BM/Hitachi 911 and on the comparison instruments using one control serum (see table 10, appendix). Results were defined as being acceptable if their deviations from the target values did not exceed by more than ±5% for classical analytes and ±10% for the homogeneous immunoassays.

As shown in figure 10, all values measured on BM/Hitachi 911 fulfilled the acceptance criteria except cholesterol, iron and uric acid. Decreased values were found for cholesterol and iron in two laboratories on BM/Hitachi 911, whereas acceptable results were obtained on the comparison instruments. Slightly increased uric acid values (+6%) were measured in one of three laboratories on BM/Hitachi 911.

Similar results were obtained in the quality control study

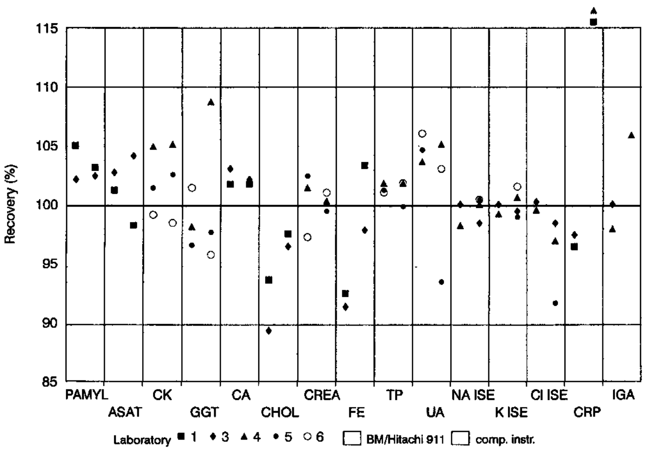

*Figure 10. Interlaboratory survey using a control serum with concentrations unknown to the evaluators. Each symbol represents the median from measurements performed over 10 days in one laboratory. Results on BM/Hitachi 911 (dashed bars) and on the comparison instrument (open bars) are shown.*

with three control sera (see table 11, appendix). In addition, the recovery of pancreatic amylase in one control (106·6%) exceeded the predefined limit.

In the quality control study, recovery experiments with the CEDIA® assays for therapeutic drug monitoring, and with urine as sample material, were also included. The recovery of phenobarbital and phenytoin was within the acceptance criterion of $\pm 10\%$ at the target value, whereas that of theophylline was 88% in one control serum with a concentration below the therapeutic range, in one of three laboratories on both BM/Hitachi 911 and on the comparison instrument. This effect was not confirmed in a satellite study in which a recovery of 98·4% was obtained using the same control material.

In control urines, all analytes met the acceptance criteria, except creatinine, for which, in one control urine, a recovery of 106·9% was obtained on BM/Hitachi 911 and of 110·4% on the comparison instrument. In the other two control urines, the recovery on BM/Hitachi 911 was between 90% to 95%.

### *Method comparison*

In total, 74 method comparison studies were performed using fresh human sera or urines; the resulting regression equations are shown in the appendix (table 12). All method comparisons were presented graphically and assessed by the evaluators.

For the determination of enzymes and substrates in serum and urine, agreement with the comparison method was rated as being sufficient if the slope of the regression equation did not deviate more than 5% from unity and the intercept was less than 5% of the diagnostically important decision levels. The acceptance criteria were increased to $\pm 10\%$ slope deviation for the homogeneous immunoassays. Depending on the analyte, either the detection limit or a deviation of $\pm 10\%$ from the reference value were tolerated as intercept for these assays.

Assessment of electrolyte concentrations in serum is especially critical. Due to the narrow physiological range of these analytes, a relatively large confidence interval for the regression line was obtained. Therefore, method comparisons were judged by the concentration range in which the difference between the methods was less than 5%. The comparisons were accepted if less than 5% deviations were obtained within the following concentration ranges:

(1) Sodium 120–170 mmol/l

(2) Potassium 2– 10 mmol/l

(3) Chloride 80–130 mmol/l.

As examples, six method comparisons comprising three constituents of serum (creatine kinase, iron, potassium), one of urine (sodium) and two homogeneous immunoassays (one turbidimetric protein assay and one CEDIA® assay) are shown in figure 11.

Method comparisons not fulfilling the acceptance criteria are listed in table 8. For classical clinical chemistry analytes in serum, seven comparisons out of 33 did not meet the acceptance criteria. Two out of 12 method comparisons in urine were unacceptable. Comparisons of homogeneous immunoassays included seven protein assays and 13 drug assays. All method comparisons fulfilled the acceptance criteria except one for digoxin.

One of the 12 method comparisons for electrolytes in serum (sodium) did not meet the acceptance criteria. The range in which the results obtained on BM/Hitachi 911 and on the comparison instrument deviated less than 5% was 127–194 mmol/l instead of the required concentration range of 120–170 mmol/.

### *Reliability*

Reliability during the evaluation phase was rated with the aid of a logbook in which any breakdown, defect, malfunction or incident of the analysis system was recorded. Some technical problems occurred during the evaluation.

The ISE unit at most evaluation sites had problems, for example noise alarm, air bubbles and crystallization of potassium chloride at the sipper probe of the dilution vessel. These resulted in an increased imprecision. A major modification of the ISE unit at the beginning of the main trial, which caused some delay in several laboratories, improved the performance.

No other systematic malfunctions were observed. Single malfunction episodes occurred with the sample probe and with the stirrer resulting in both situations in a wrong home position and a stoppage of the analyser. Software malfunctions reported during the multicentre evaluation were corrected in two new system software releases.

### *Assessment of practicability*

The practicability of BM/Hitachi 911 was judged in comparison with the present situation in the individual laboratories. The median of all laboratories was calculated from the mean of all scores obtained for each group of attributes. These results are shown in figure 12.

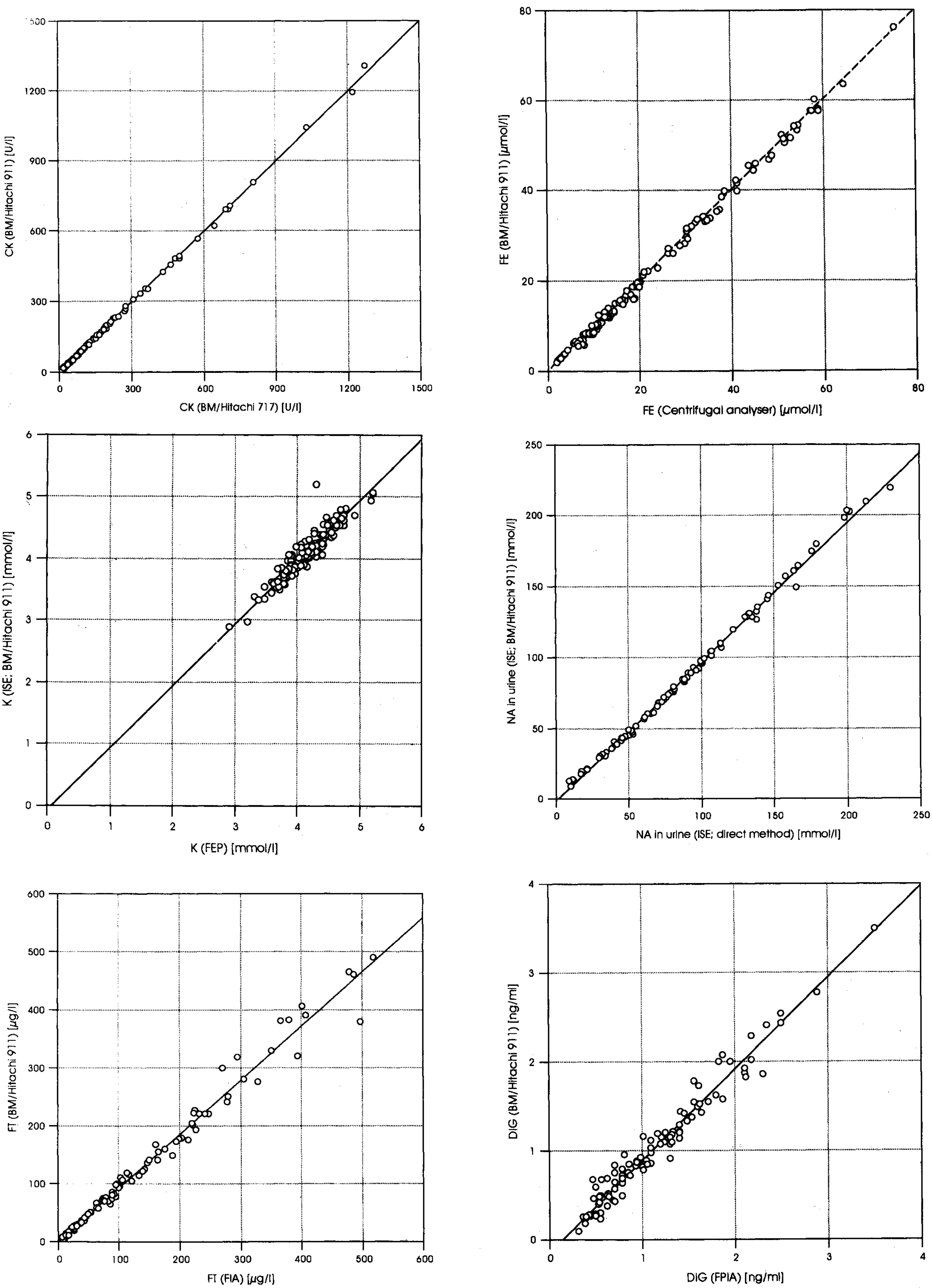

*Figure 11. Method comparison for some of the analytes in human serum/plasma/urine analysed on BM/Hitachi 911 and comparison instruments. Solid lines represent regression equations calculated according to Passing and Bablok [10] and dotted lines $y = x$. The following regression equations were obtained: CK ($N = 150$), $y = 1{\cdot}00x + 0{\cdot}0$; FE ($N = 150$) $y = 1{\cdot}00x - 0{\cdot}15$; K ($N = 150$) $y = 1{\cdot}00x - 0{\cdot}07$; NA in urine ($N = 100$) $y = 0{\cdot}98x - 1{\cdot}35$; FT ($N = 100$) $y = 0{\cdot}93x + 0{\cdot}33$; DIG ($N = 100$) $y = 1{\cdot}03x - 0{\cdot}14$.*

*Table 8. Individual method comparison results exceeding the acceptance limits.*

| Analyte | Unit | Regression anlysis | | Comparison method/instrument |
|---|---|---|---|---|
| | | Slope | Intercept | |
| γ-Glutamyltransferase | U/l | 0·91 | 0·20 | BM/Hitachi 737 |
| Calcium | mmol/l | 1·06 | −0·11 | Atomic absorption spectroscopy |
| Cholesterol | mmol/l | 1·08 | −0·33 | BM/Hitachi 704 |
| Creatinine | µmol/l | 1·11 | −8·81 | BM/Hitachi 717 |
| Iron | µmol/l | 0·92 | 0·17 | BM/Hitachi 704 |
| Iron | µmol/l | 0·93 | 0·37 | BM/Hitachi 747 |
| Fructosamine | µmol/l | 0·98 | −17·35 | Reduction of nitroblue tetrazolium chloride; longer incubation time |
| Albumin in urine | mg/l | 1·60 | −2·46 | Rate nephelometry |
| Albumin in urine | mg/l | 0·73 | −1·50 | Radioimmunoassay |
| Digoxin | ng/ml | 0·75 | −0·06 | Fluorescence polarization immunoassay |

When the medians of the scores for BM/Hitachi 911 were compared with those of the present laboratory situation, 12 of 14 groups of attributes were rated higher for the new analysis system. Especially high scores were given for spatial arrangement, sample processing, reagent handling, workflow, calibration and versatility. The medians of the scores for training/operation and maintenance/troubleshooting were identical for BM/Hitachi 911 and the comparison instruments. None of the attributes was rated lower on BM/Hitachi 911.

More detailed information on the distribution of scores in relation to the main topics is shown in figure 13. Higher scores (8 to 10) were given more frequently for BM/Hitachi 911 than for the existing laboratory situation. For BM/Hitachi 911, out of 194 attributes 12 were rated with a score of 0 to 1 (each by only one evaluation site) and 93 attributes with a score of 9 or 10 (each by one or several laboratories).

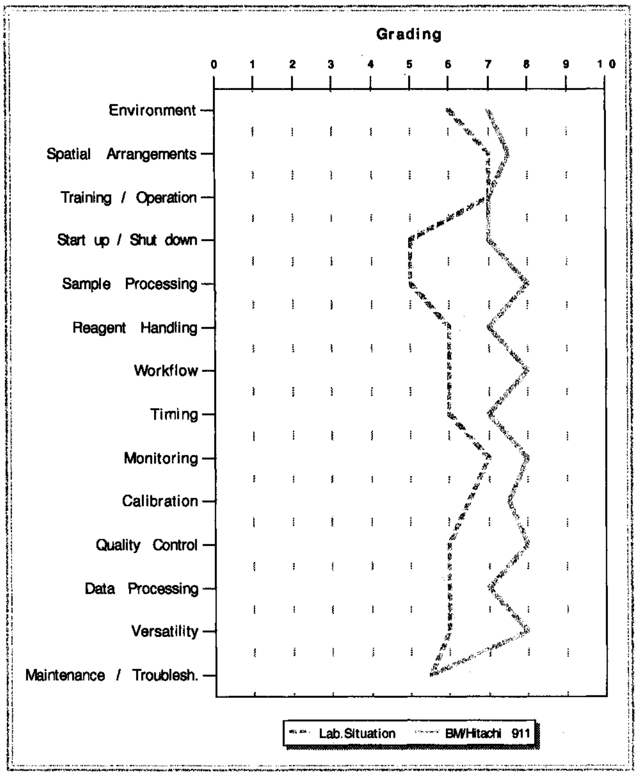

*Figure 12. Assessment of practicability of the BM/Hitachi 911 analysis system (solid line) compared to the laboratory situation (dashed line). The median of the scores is shown.*

### Discussion

The versatility of the BM/Hitachi 911 analysis system was tested in the multicentre study in different laboratory sections—routine, STAT, homogeneous immunoassays and urinalysis. The results were assessed on the basis of quality specifications for the various performance characteristics previously agreed upon by the group of evaluators. A good performance was found for most of the analytes in all laboratory sections using different sample materials of serum, plasma and urine. Although some analytes did not fulfill one or the other acceptance criterion, none could be rated unacceptable. The results outside the acceptance criteria are discussed below with the exception of the enzymatic methods for determination of sodium, potassium and chloride. The performance of these methods was unacceptable on the BM/Hitachi 911. New standardization procedures for these tests are under development.

### Within-run imprecision

The results for control and human materials (see table 5) show that overall the instrument had given a good performance. The individual CVs of a few analytes were slightly higher than the acceptance limits. These were found mainly in one laboratory and in one control material. Variable results were obtained for urinary albumin in a control material with a concentration below

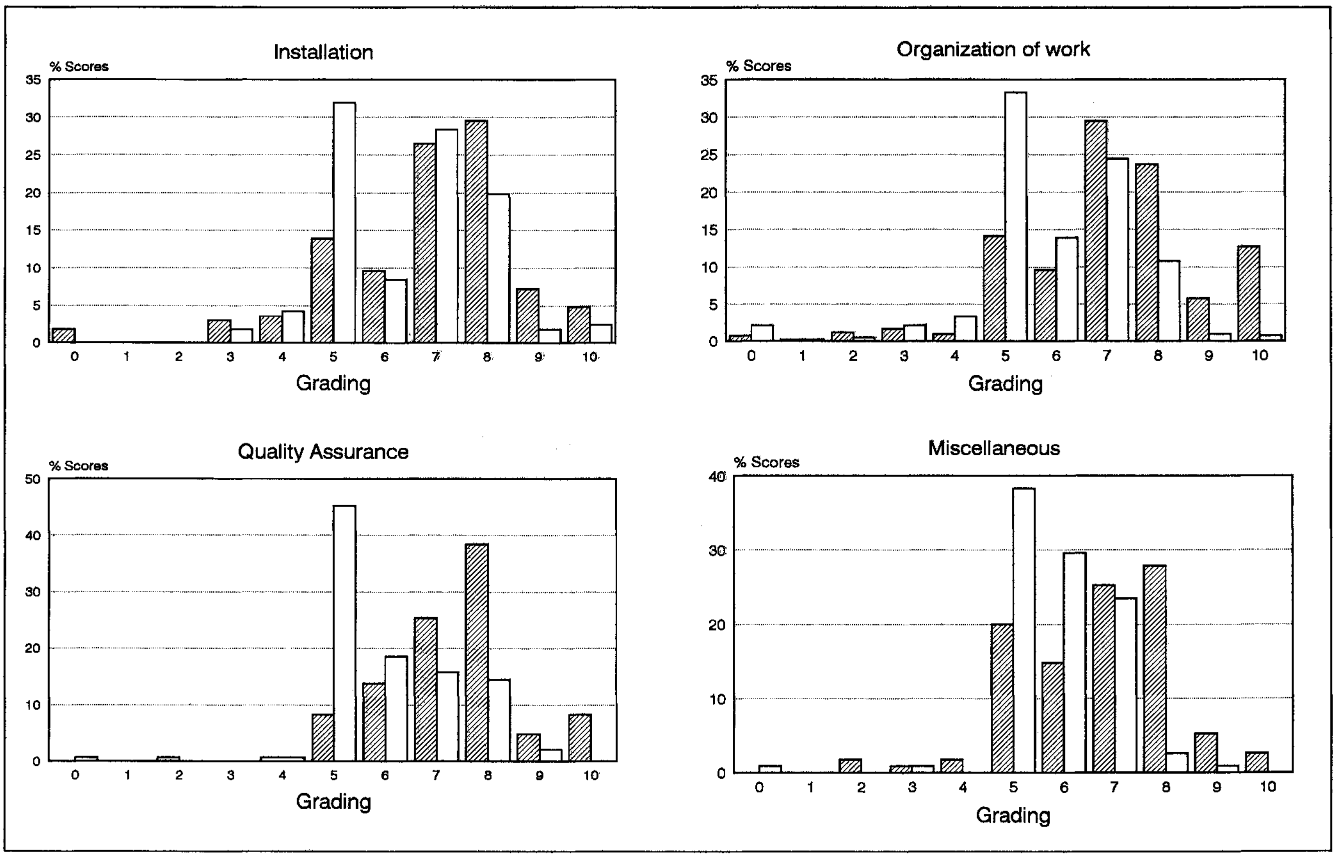

*Figure 13. Assessment of practicability. Distribution of scores for the main groups installation, organization of work, quality assurance and miscellaneous. Dashed bars represent the grading for the BM/Hitachi 911 and open bars that of the present laboratory situation.*

the decision level (CV values of 2·1, 2·2 and 9·2% at 15 mg/l). This variation in results could not be reproduced in a human urine pool with an albumin concentration of 33 mg/l. With this material, a CV of 1·4% was obtained (see table 5).

*Between-day imprecision*

Individual CV values of several analytes exceeded the acceptance limits. For calcium, cholesterol and creatinine the maximum CVs of 3·1 and 3·3% may still be seen as borderfline cases. The maximum CVs of 3·7% for sodium and 2·3% for chloride reflected the condition of the ISE unit which had to be modified during the evaluation. In spite of this modification, an improvement was not observed in all laboratories. In one laboratory, the CEDIA® Phenytoin and Digoxin assays, and the urinary albumin assay in the low level control, also showed CV values slightly higher than 10%.

*Quality specifications*

Comparing the between-day imprecision results of the BM/Hitachi 911 with the proposed quality specifications for the imprecision of analytical systems for clinical chemistry [12, 13], it was concluded that the BM/Hitachi 911 analysis system achieved these specifications for nearly all analytes evaluated in the study (table 9). The proposed quality specifications were not met with the calcium assay (interim specification 1·5%, BM/Hitachi 911 1·6%), creatinine assay (specification 2·2%, BM/Hitachi 911 2·3%), sodium assay (interim specification 0·7%, BM/Hitachi 911 1·4%) and the chloride assay (interim specification 1·0%, BM/Hitachi 911 1·7%). However, since the imprecision of the calcium and the creatinine assay were only 0·1% higher than the specifications, the results obtained can be judged acceptable. Owing to the low biological variation of sodium and chloride, the imprecision specifications were set very low: 0·3% for sodium and 0·7% for chloride. Since no available technology is capable of producing such a low imprecision at a reasonable cost, interim specifications with 0·7% and 1·0% were proposed. Even these values are unlikely to be easily achieved with the existing technology.

*Analytical range limits*

The acceptance criteria for linearity of the measuring range were fulfilled for all analytes, except for urinary albumin at concentrations below 25 mg/l. This non-linearity resulted in an underestimation of about 3 mg/l albumin at the decision level of 20 mg/l. This is within the range found during the multicentre evaluation for the albumin test [14]. In view of the large biological variation of this analyte, this deviation was considered acceptable. During the evaluation, single-point calibration had been used for the C-reactive protein assay. This mode of

*Table 9. Comparison of quality specifications for between-day imprecision and inaccuracy proposed by Fraser et al. [12 and 13] with the results of the BM/Hitachi 911; in parentheses interim specifications. The data of BM/Hitachi 911 were obtained with Precinorm® U, except for TF, IGA (Precinorm® Protein) and drugs (Precinorm® TDM level 3).*

| Analyte | Quality specifications between-day CV (%) | BM/Hitachi 911 between-day CV (%) | Quality specifications inaccuracy (% deviation) | BM/Hitachi 911 mean recovery (% deviation) |
|---|---|---|---|---|
| PAMYL | 3·7 | 1·5 | 6·5 | 6·6 |
| ASAT | 7·2 | 1·9 | 6·2 | 3·4 |
| CK | 20·7 | 1·3 | 19·8 | 1·9 |
| GGT | 7·4 | 2·3 | 21·8 | 2·6 |
| CA | 0·9 (1·5) | 1·6 | 0·7 (1·8) | 1·8 |
| CHOL | 2·7 | 1·6 | 4·1 | 6·7 |
| CREA | 2·2 | 2·3 | 2·8 (4·4) | 1·1 |
| FE | 15·9 | 2·1 | 8·9 | 4·5 |
| TP | 1·4 | 1·1 | 1·5 (2·8) | 1·4 |
| UA | 4·2 | 1·3 | 4·0 (8·4) | 5·9 |
| NA | 0·3 (0·7) | 1·4 | 0·2 (0·6) | 0·5 |
| K | 2·4 | 1·8 | 1·6 (4·8) | 1·0 |
| CL | 0·7 (1·0) | 1·7 | 0·5 (1·4) | 0·6 |
| TF | 2·4 (4·0) | 1·5 | 2·3 (4·8) | 5·4 |
| IGA | 2·2 (3·8) | 2·9 | 12·5 | 3·2 |
| DIG | 3·8 (4·7) | 3·7 | 3·9 | 1·6 |
| THEO | 11·1 | 2·4 | 5·4 | 2·8 |
| PHEBA | 2·2 | 2·2 | 5·6 | 2·0 |
| PHENY | 3·6 | 3·3 | 4·1 | 4·3 |

calibration gave an upper limit of the analytical range of 80 mg/l which is not sufficient for diagnostic purposes. However, studies performed during the multicentre evaluation of the C-reactive protein assay using multi-point calibration showed a higher measuring range at least up to 250 mg/l [15], or even higher, depending on the standards used. Therefore, only multi-point calibration is recommended now by the manufacturer, and the results obtained during this evaluation are not reported.

### *Interferences*

Increased total protein values were measured at haemoglobin concentrations above 4 g/l. This can be readily explained by the fact that haemoglobin is a protein. The activity of γ-glutamyltransferase was decreased in haemolytic samples. As a consequence, the γ-glutamyltransferase test will be modified by the manufacturer in order to reduce this interference. Increased activities of creatine kinase, and especially creatine kinase MB isoform, were found in the presence of haemoglobin. This is caused by the release of adenylate kinase from erythrocytes. Again, the manufacturer intends to modify the application for the creatine kinase MB isoform assay, with the aim of reducing the susceptibility of the method to interference by haemoglobin.

Increased immunoglobulin A concentrations had been measured in lipaemic samples. Therefore, a modified immunoglobulin A assay is presently being developed to overcome this problem. The effect of turbidity on aspartate aminotransferase activities has previously been observed during other BM/Hitachi system evaluations [3]. It can be explained by the fact that the fresh reagents for this assay have high initial absorbance and that the addition of a turbid specimen would cause the total absorbance to exceed the photometric measurement range of 3·3 absorbance units.

### *Accuracy*

During the interlaboratory survey and the quality control study a recovery of less than 95% was found for cholesterol. The assigned value of this analyte was confirmed by the reference method of isotope dilution/mass spectrometry [16]. Therefore, the effect could not be attributed to a wrongly assigned value. In two of the three method comparison studies a good agreement was found, whereas in the third increased cholesterol values were obtained on BM/Hitachi 911. These contradictory results could not be explained. The effect was not observed in a satellite study performed in one of the evaluation centres.

Iron values below the acceptance criteria were found in three of the four control sera used in the interlaboratory survey and the quality control study. The effect was confirmed in the method comparison study in two of the three laboratories (table 8). In the third laboratory, in which the comparison instrument was a centrifugal analyser, a slope of 1·00 and an intercept of −0·15 μmol/l iron were obtained in the regression analysis. The deviant results were probably caused by wrongly low values being assigned to the calibrators of the two comparison instruments.

If the results of the accuracy experiments are compared to the quality specifications proposed by Fraser *et al.* [12, 13], all assays performed on BM/Hitachi 911 met the requirements with the exception of cholesterol and transferrin.

Eleven out of 74 method comparisons gave slope intervals and intercepts that were outside the acceptance limits

(table 8). Differences in the $\gamma$-glutamyltransferase assay were caused by an incorrect temperature conversion factor on the comparison instrument in one laboratory. The method comparison for the calcium assay yielded a slope of 1·06. However, the differences between the values of individual samples (from $-0·13$ to 0·18 mmol/l) were acceptable. In one of three laboratories a slope of 1·11 was obtained in the method comparison for creatinine. In spite of this, agreement was present at the decision level. However, with this high slope, results of samples from dialysis patients are not correctly interpreted. In a satellite study in one of the laboratories, the deviation was confirmed. The manufacturer will change the application in order to correct the discrepancy. The lack of agreement in the frustosamine assay could be explained by the fact that the comparison method was performed with a longer incubation time. The evaluators accepted this deviation.

Albumin in urine showed discrepant results in two of the three laboratories where the assay was compared with nonturbidimetric methods. One out of the four digoxin method comparisons was outside the accepted range. The experiment had been performed in a laboratory where many samples were derived from haemodialysis patients. These samples might contain digoxin-like immunoreactive factors which are known to interfere digoxin assays [17].

The results of the C-reactive protein method comparisons are not reported because single-point calibration used in the experiments had much narrower linear range than was originally thought. Therefore, the application was later changed to multi-point calibration.

### *Practicability*

The outstanding feature of the BM/Hitachi 911 analysis system is that it can be used as a consolidated workstation for assays from diverse segments of a clinical laboratory (clinical chemistry, proteins, therapeutic drug monitoring). All tests necessary for a complete patient profile can be performed using one primary tube without sample splitting and additional manual workload. This simplifies the distribution of samples and thus the organization of the laboratory. Hazards of sample mix-up as well as cost for sample splitting are avoided. Moreover, analyses requiring a predilution step (for example, immunoglobulins) can also be handled easily without the need for high sample volume. The analyser performs the predilution itself.

Urine samples can be run randomly in series with serum or plasma specimens on BM/Hitachi 911. No relevant carry-over effects between the different sample materials were observed during the multicentre evaluation. An excellent analytical performance was obtained for urine samples, showing that the instrument is well suited for urinalysis.

Suitability of BM/Hitachi 911 for routine analyses depends, of course, on the size of the laboratory. One of the evaluators considered the instruments well suited for the routine requirements of his laboratory. The STAT facilities were rated positively in all evaluation centres.

The acceptance criteria for the analytical performance of the Boehringer Mannheim/Hitachi 911 analysis system were fulfilled for all laboratory segments with only a few exceptions. Moreover, the practicability of this instrument exceeded the requirements for most of the attributes. Owing to its versatility, the instrument is best placed as a consolidated workstation in the small- to medium-sized laboratory, or as a back-up instrument for special determinations like proteins, drugs or urinalysis in a large laboratory.

### Acknowledgement

The authors wish to thank all their co-workers in the respective laboratories and departments participating in the study for their excellent support.

### References

1. ECCLS, Guidelines for the evaluation of analysers in clinical chemistry. *ECCLS Document*, **3** (1986), 2
2. Bablok, W., Barembruch, R., Stockman, W., Brauer, P., Graber, P., Michel, R and Vonderschmidtt, D., *Journal of Automatic Chemistry*, **13** (1991), 167.
3. Bonini, P., Ceriotti, F., Keller, F., Brauer, P., Stolz, H., Pascual, C., García Beltrán, L., Vonderschmidtt, D. J., Pei, P., Bablok, W., Domke, I. and Stockmann, W., *European Journal of Clinical Chemistry and Clinical Biochemistry*, **30** (1992), 881.
4. Bayer, P. M., Knedel, M., Montalbetti, N., Brenna, S., Prencipe, L., Vassault, A., Bailly, M., Phung, H. T., Bablok, W., Poppe, W. and Stockmann, W., *European Journal of Clinical Chemistry and Clinical Biochemistry*, **25** (1987), 919.
5. Baadenhuijsen, H., Bayer, P. M., Keller, H., Knedel, M., Montalbetti, N., Brenna, S., Prencipe, L., Vassault, A., Bailly, M., Phung, H. T., Bablok, W., Poppe, W. and Stockmann, W., *European Journal of Clinical Chemistry and Clinical Biochemistry*, **28** (1990), 261.
6. Stockmann, W., Bablok, W., Poppe, W., Bayer, P. M., Keller, F. and Schweiger, C. R., in *Evaluation Methods in Laboratory Medicine*, Ed. Haeckel, R. (VCH, Weinheim, 1993), 185.
7. Bablok, W., in *Evaluation Methods in Laboratory Medicine*, Ed. Haeckel, R. (VCH, Weinheim, 1993), 251.
8. Glick, M. R., Ryder, K. W. and Jackson, S. A., *Clinical Chemistry*, **32** (1986), 470.
9. Bablok, W., Haeckel, R., Meyers, W. and Wosniok, W., in *Evaluation Methods in Laboratory Medicine*, Ed. Haeckel, R. (VCH, Weinheim, 1993), 203.
10. Passing, H. and Bablok, W., *European Journal of Clinical Chemistry and Clinical Biochemistry*, **21** (1983), 709.
11. Broughton, P. M. G., Gowenlock, A. H., McCormack, J. J. and Neill, D. W., *Annals of Clinical Biochemistry*, **11** (1974), 207.
12. Fraser, C. G., Hyltoft Peterson, P., Ricos, C. and Haeckel, R., *European Journal of Clinical Chemistry and Clinical Biochemistry*, **30** (1992), 311.
13. Fraser, C. G., Hyltoft Petersen, P., Ricos, C. and Haeckel, R., in *Evaluation Methods in Laboratory Medicine*, Ed. Haeckel, R. (VCH, Weinheim, 1993), 87.
14. Hubbuch, A., *Wiener Klinische Wochenschrift*, **103**, Suppl. 189 (1991), 23.
15. Borque de Larrea, L., Rus, A., Carlström, A., Pira, U., Delobbes, E., Thiry, P., Ferrari, L., Barozzi, D., Hafner, G., Lotz, J., van Oers, R. J. M., Leerkes, B., Szymanowicz, A., Dubois, H., Hallstein, A. and Domke, I., *Klin. Lab.*, **39** (1993), 55.
16. Cohen, A., Hertz, H. J., Mandel, J., Pank, R. C., Schaffer, R., Sniegoski, L. T., Sun, T., Welch, M. J. and White, V. E., *Clinical Chemistry*, **26** (1980), 854.
17. Schlebusch, H., Jarausch, J. and Domke, I., *Wiener Klinische Wochenschrift*, **104**, Suppl. 191 (1992), 59.

### Appendix

*Table 10. Control and calibration materials.*

| Calibrator/control material | Lot No. |
|---|---|
| Calibrator for automated systems, C.f.a.s. (lyophilized) | 172 219 |
| Calibrator for automated systems, C.f.a.s (liquid)* | 176 286 |
| ISE Compensator | 636 238 |
| Precimat® Fructosamine | 172 078 |
| Preciset® MAU | 176 872 |
| Precimat® BNAG | 628 755 |
| Precimat® CRP | 17463802 |
| Precimat® IGA | 17420001 |
| Precimat® Transferrin | 172644701 |
| Preciset® Ferritin | 173 223 |
| Calibrator low/high THEO | A 7601 C/A 7602 C |
| Calibrator low/high PHEBA | A 7735 B/A 7736 B |
| Calibrator low/high PHENY | A 7502 B/A 7511 B |
| Calibrator low/high DIG | A 7667 B/A 7668 B |
| Precinorm® U | 169 151 |
| Precipath® U | 173 579 |
| Precipath® Evaluation* | 174 412 |
| Precinorm® Fructosamine | 175 900 |
| Precipath® Fructosamine | 171 630 |
| Precinorm® MAU | 175 221 |
| Precipath® MAU | 175 367 |
| Precinorm® NAG | 628 757 |
| Precinorm® Protein | 177 031 |
| Precinorm® TDM 1 | 17505090 |
| Precinorm® TDM 3 (Boehringer Mannheim GmbH, Mannheim, Germany) | 17505290 |
| Interlaboratory survey | |
| Precinorm® U | 168 588 |
| C.f.a.s. Proteins | 178 586 |
| Lyphochek® | |
| Quantitative urine control normal level (Bio-Rad Laboratories, München, Germany) | 52201 |

* Only for evaluation purposes.

*Table 11. Imprecision and recovery of all laboratories in individual control materials on the BM/Hitachi 911.*

| Analyte [unit] | Control material | Assigned value | Between-day Mean recovery Median | Between-day Mean recovery Range | Between-day CV% Median | Between-day CV% Range | Within-run CV% Median | Within-run CV% Range |
|---|---|---|---|---|---|---|---|---|
| PAMYL [U/l] | Precinorm® U | 385 | 106·6 | 104·5–107·0 | 1·5 | 1·5–2·9 | 0·6 | 0·6–1·2 |
| | Precipath® U | 518 | 102·4 | 99·2–103·1 | 1·1 | 1·0–2·9 | 0·8 | 0·7–0·8 |
| | Precipath® Evaluation | 504 | 100·4 | 99·3–102·0 | 1·7 | 1·3–2·4 | 1·0 | 0·8–1·1 |
| ASAT [U/l] | Precinorm® U | 53 | 103·4 | 97·3–104·6 | 1·9 | 1·3–2·7 | 1·6 | 1·2–2·2 |
| | Precipath® U | 125 | 100·5 | 94·8–100·7 | 1·4 | 1·0–1·5 | 0·8 | 0·8–0·9 |
| | Precipath® Evaluation | 90 | 100·9 | 97·8–103·0 | 1·8 | 1·5–2·1 | 1·3 | 1·2–1·4 |
| CK [U/l] | Precinorm® U | 258 | 98·1 | 97·4–103·5 | 1·3 | 1·0–1·3 | 0·9 | 0·8–1·2 |
| | Precipath® U | 471 | 98·7 | 98·1–103·8 | 1·2 | 0·7–1·2 | 0·7 | 0·5–1·6 |
| | Precipath® Evaluation | 322 | 102·2 | 100·6–106·8 | 1·2 | 0·8–1·5 | 1·1 | 0·7–1·6 |
| GGT [U/l] | Precinorm® U | 62 | 97·4 | 95·5– 98·2 | 2·3 | 1·6–2·4 | 1·1 | 1·0–1·4 |
| | Precipath® U | 189 | 99·0 | 97·4–101·0 | 1·7 | 1·4–2·0 | 0·5 | 0·5–0·8 |
| | Precipath® Evaluation | 102 | 100·7 | 98·2–102·5 | 1·7 | 1·5–1·7 | 0·8 | 0·7–1·0 |

(*continued*)

*Table 11 (continued).*

| Analyte [unit] | Control material | Assigned value | Between-day Mean recovery Median | Between-day Mean recovery Range | Between-day CV% Median | Between-day CV% Range | Within-run CV% Median | Within-run CV% Range |
|---|---|---|---|---|---|---|---|---|
| CA | Precinorm® U | 2·3 | 101·8 | 100·4–103·5 | 1·6 | 1·4– 2·7 | 1·4 | 0·7–1·5 |
| [mmol/l] | Precipath® U | 3·5 | 100·0 | 99·7–102·1 | 2·0 | 1·4– 2·5 | 0·8 | 0·7–0·8 |
| | Precipath® Evaluation | 2·4 | 101·3 | 101·3–101·3 | 1·7 | 1·4– 3·1 | 0·8 | 0·8–1·5 |
| CHOL | Precinorm® U | 2·9 | 93·3 | 87·7– 95·5 | 1·6 | 1·2– 2·6 | 1·6 | 1·2–1·9 |
| [mmol/l] | Precipath® U | 3·2 | 89·3 | 80·3– 91·8 | 2·6 | 2·3– 3·1 | 1·6 | 1·1–1·8 |
| | Precipath® Evaluation | 4·0 | 96·1 | 92·9– 98·5 | 2·1 | 1·5– 2·4 | 2·0 | 1·5–2·1 |
| CREA | Precinorm® U | 190 | 101·1 | 100·1–101·4 | 2·3 | 1·5– 2·5 | 1·6 | 0·9–1·6 |
| [μmol/l] | Precipath® U | 344 | 98·3 | 97·1–100·2 | 2·7 | 1·7– 3·1 | 1·0 | 0·8–1·5 |
| | Precipath® Evaluation | 163 | 94·8 | 94·4– 96·0 | 2·9 | 1·7– 3·3 | 1·4 | 1·4–1·5 |
| FRUCT | Precinorm® Fructosamine | 287 | 96·8 | | 3·1 | | 1·0 | 0·7–1·1 |
| [μmol/l] | Precipath® Fructosamine | 552 | 101·1 | | 2·0 | | 0·9 | 0·8–1·0 |
| FE | Precinorm® U | 19·5 | 85·5 | 84·7– 86·2 | 2·1 | 1·4– 2·2 | 1·8 | 1·3–3·1 |
| [μmol/l] | Precipath® U | 24·6 | 94·6 | 92·8– 95·9 | 2·2 | 2·0– 2·3 | 1·3 | 0·9–1·5 |
| | Precipath® Evaluation | 32·7 | 97·1 | 96·5– 98·2 | 1·9 | 1·3– 2·5 | 0·9 | 0·9–1·5 |
| TP | Precinorm® U | 51 | 101·4 | 100·6–102·4 | 1·1 | 1·0– 1·1 | 0·5 | 0·4–1·5 |
| [g/l] | Precipath® U | 42 | 105·0 | 103·8–106·0 | 1·4 | 1·1– 1·5 | 0·8 | 0·4–1·1 |
| | Precipath® Evaluation | 52 | 104·1 | 102·9–104·5 | 1·4 | 1·2– 1·5 | 0·7 | 0·5–1·4 |
| UA | Precinorm® U | 289 | 105·9 | 105·6–108·3 | 1·3 | 1·2– 2·6 | 0·6 | 0·4–0·7 |
| [μmol/l] | Precipath® U | 589 | 102·5 | 102·4–104·6 | 1·3 | 1·0– 1·7 | 0·5 | 0·5–0·8 |
| | Precipath® Evaluation | 247 | 103·8 | 103·2–106·9 | 1·5 | 1·3– 2·9 | 1·0 | 0·9–1·0 |
| NA | Precinorm® U | 129 | 99·5 | 98·2–101·3 | 1·4 | 1·4– 1·4 | 0·7 | 0·4–1·0 |
| [mmol/l] | Precipath® U | 135 | 100·1 | 100·1–101·7 | 1·4 | 1·3– 1·9 | 0·4 | 0·3–1·0 |
| | Precipath® Evaluation | 84 | 101·7 | 96·4–101·7 | 2·6 | 2·3– 3·7 | 1·0 | 0·5–1·3 |
| K | Precinorm® U | 4·9 | 99·0 | 98·5–101·0 | 1·8 | 1·0– 2·0 | 0·8 | 0·3–1·1 |
| [mmol/l] | Precipath® U | 6·6 | 100·8 | 100·1–102·4 | 1·7 | 1·5– 1·9 | 0·6 | 0·4–1·4 |
| | Precipath® Evaluation | 4·6 | 101·1 | 100·5–101·5 | 1·6 | 1·3– 2·0 | 0·7 | 0·5–1·3 |
| CL | Precinorm® U | 85 | 100·6 | 98·0–101·2 | 1·7 | 1·6– 2·3 | 1·1 | 0·5–1·2 |
| [mmol/l] | Precipath® U | 105 | 103·5 | 100·5–104·6 | 1·7 | 1·6– 2·3 | 0·5 | 0·4–0·7 |
| | Precipath® Evaluation | 101 | 96·4 | 95·0– 97·3 | 1·8 | 1·6– 2·3 | 0·7 | 0·6–0·8 |
| CRP [mg/l] | Precinorm® Protein | 49 | 102·7 | 100·7–104·6 | 2·6 | 0·8– 4·7 | 1·3 | 0·8–1·8 |
| IGA | Precinorm® U | 1·8 | | 96·9– 97·8 | | 1·3– 2·3 | | 1·9–2·0 |
| [g/l] | Precinorm® Protein | 3·6 | | 96·8– 96·8 | | 0·7– 1·5 | | 0·9–1·4 |
| FT [μg/l] | Precinorm® Protein | 95 | 102·0 | | 2·8 | | 1·6 | 1·3–3·5 |
| TF | Precinorm® U | 2·3 | 98·0 | | 6·9 | | 3·0 | 2·0–3·0 |
| [g/l] | Precinorm® Protein | 4·5 | 94·6 | | 2·9 | | 1·5 | 1·4–1·9 |
| PHEBA | Precinorm® TDM 1 | 6·1 | 103·3 | 96·7–103·3 | 6·0 | 3·1– 6·4 | 3·9 | 2·9–4·2 |
| [μg/ml] | Precinorm® TDM 3 | 49·2 | 97·8 | 95·6–102·0 | 2·2 | 1·1– 2·9 | 1·7 | 1·7–1·8 |
| PHENY | Precinorm® TDM 1 | 4·4 | 106·8 | 95·5–106·8 | 6·6 | 4·6–11·7 | 5·9 | 5·6–8·4 |
| [μg/ml] | Precinorm® TDM 3 | 25·4 | 95·7 | 94·9– 96·1 | 3·3 | 2·3– 4·0 | 2·6 | 2·1–4·5 |
| THEO | Precinorm® TDM 1 | 5·0 | 94·0 | 88·0– 96·0 | 4·2 | 3·6– 5·7 | 4·8 | 4·3–4·8 |
| [μg/ml] | Precinorm® TDM 3 | 25·3 | 97·2 | 93·7– 98·4 | 2·4 | 2·4– 2·5 | 2·1 | 2·0–2·3 |
| DIG | Precinorm® TDM 1 | 0·95 | 101·1 | 91·6–110·5 | 9·4 | 6·9–10·2 | 7·6 | 6·5–8·8 |
| [ng/ml] | Precinorm® TDM 3 | 3·2 | 98·4 | 95·0–104·5 | 3·7 | 1·6– 4·3 | 3·5 | 2·4–4·0 |
| β-NAG [U/l] | Precinorm® BNAG | | | | 1·1 | | 0·9 | 0·8–1·1 |
| CREA | Lyphochek* 1 | 7·7 | | 101·4–106·9 | | 1·6– 3,7 | | 1·3–1·7 |
| (Urine) | Precinorm® MAU | 16·1 | | 90·7– 92·8 | | 0·9– 3·3 | | 1·1–1·2 |
| [mmol/l] | Precipath® MAU | 4·3 | | 93·5– 94·9 | | 2·7– 4·0 | | 1·3–1·4 |

*Table 11 (continued).*

| Analyte [unit] | Control material | Assigned value | Between-day Mean recovery Median | Between-day Mean recovery Range | Between-day CV% Median | Between-day CV% Range | Within-run CV% Median | Within-run CV% Range |
|---|---|---|---|---|---|---|---|---|
| MAU (Urine) [mg/l] | Precinorm® MAU | 15·5 | 90·9 | 89·7– 94·2 | 3·3 | 2·7–11·3 | 2·2 | 2·1–9·2 |
| | Precipath® MAU | 94·8 | 96·7 | 93·3– 97·8 | 1·6 | 0·8– 3·5 | 1·1 | 0·8–2·0 |
| NA (Urine) [mmol/l] | Lyphochek® 1 | 60 | 99·8 | | 1·6 | | 0·4 | 0·4–0·5 |
| K (Urine) [mmol/l] | Lyphochek® 1 | 23 | 103·3 | | 1·7 | | 0·2 | 0·2–0·3 |
| CL (Urine) [mmol/l] | Lyphochek® 1 | 53 | 94·8 | | 1·2 | | 0·4 | 0·4–0·5 |

*Table 12. Method comparison for human serum/plasma/urine analytes assayed on BM/Hitachi 911 (y) and the comparison instrument (x). Each line represents the result from one laboratory.*

| Analyte (Unit) | Sample material | $N$ | Range (from $x$) | Regression analysis $y = bx + a$: $b$ | $a$ | 95% Median distance [9] |
|---|---|---|---|---|---|---|
| PAMYL (U/l) | Serum | 150 | 8 to 727 | 1·02 | −1·27 | 6·3 |
| | Plasma | 105 | 5 to 684 | 1·01 | −0·79 | 17·3 |
| | Serum | 225 | 28 to 2010 | 1·01 | −2·59 | 32·9 |
| ASAT (U/l) | Serum | 150 | 9 to 642 | 1·03 | 0·33 | 2·2 |
| | Plasma | 105 | 10 to 549 | 0·99 | −1·16 | 3·4 |
| | Serum | 211 | 10 to 620 | 1·01 | 0·24 | 9·3 |
| CK (U/l) | Serum | 146 | 3 to 763 | 1·01 | 1·45 | 5·6 |
| | Serum | 150 | 6 to 1270 | 1·00 | 0·06 | 6·1 |
| | Plasma | 150 | 5 to 1060 | 1·03 | −0·39 | 8·7 |
| CK-MB (U/l) | Serum | 150 | 3 to 116 | 0·95 | 1·02 | 2·8 |
| | Plasma | 100 | 5 to 443 | 0·99 | −0·10 | 10·7 |
| GGT (U/l) | Serum | 147 | 3 to 1170 | 0·91 | 0·20 | 3·4 |
| | Serum | 149 | 0 to 593 | 1·00 | 0·00 | 0·1 |
| | Plasma | 150 | 6 to 1225 | 1·04 | 0·43 | 15·5 |
| CA (mmol/l) | Serum | 150 | 1·5 to 2·9 | 1·06 | −0·11 | 0·09 |
| | Plasma | 105 | 2·0 to 3·0 | 0·96 | 0·06 | 0·05 |
| | Serum | 212 | 1·4 to 3·5 | 1·00 | −0·02 | 0·11 |
| CHOL (mmol/l) | Serum | 150 | 0·9 to 10·0 | 1·08 | −0·33 | 0·15 |
| | Plasma | 105 | 1·0 to 8·2 | 1·01 | −0·01 | 0·25 |
| | Serum | 150 | 1·4 to 13·9 | 1·01 | 0·00 | 0·24 |
| CREA (μmol/l) | Serum | 149 | 23·9 to 1775 | 1·04 | 0·4 | 18·0 |
| | Serum | 150 | 34·0 to 1169 | 1·11 | −8·81 | 11·5 |
| | Plasma | 150 | 47·0 to 648 | 1·02 | −6·81 | 13·1 |
| FE (μmol/l) | Serum | 150 | 1·0 to 45 | 0·92 | 0·17 | 0·5 |
| | Plasma | 90 | 0·4 to 48 | 0·93 | 0·37 | 1·1 |
| | Serum | 150 | 1·9 to 76 | 1·00 | −0·15 | 1·1 |
| FRUCT (μmol/l) | Plasma | 100 | 143 to 626 | 0·98 | −17·35 | 19·4 |
| TP (g/l) | Serum | 147 | 38 to 81 | 1·04 | −0·30 | 1·5 |
| | Serum | 150 | 42 to 117 | 1·04 | 0·02 | 2·2 |
| | Plasma | 150 | 34 to 91 | 1·05 | −1·48 | 2·7 |
| UA (μmol/l) | Serum | 149 | 59 to 987 | 1·01 | −12·03 | 20·2 |
| | Serum | 150 | 68 to 1034 | 1·05 | −16·33 | 46·6 |
| | Plasma | 150 | 76 to 834 | 1·00 | −3·83 | 30·2 |

(continued)

*Table 12* (*continued*).

| Analyte (Unit) | Sample material | *N* | Range (from *x*) | Regression analysis $y = bx + a$ *b* | *a* | 95% Median distance [9] |
|---|---|---|---|---|---|---|
| NA (mmol/l) | Plasma | 90 | 130 to 149 | 1·24 | −36·79 | 2·68 |
| | Plasma | 90 | 129 to 149 | 1·18 | −28·03 | 2·09 |
| | Serum | 75 | 119 to 152 | 1·07 | −8·75 | 2·17 |
| | Serum | 75 | 131 to 151 | 1·17 | −26·35 | 1·67 |
| K (mmol/l) | Plasma | 90 | 3·20 to 5·20 | 1·05 | −0·36 | 0·17 |
| | Plasma | 150 | 2·91 to 5·22 | 1·00 | −0·07 | 0·13 |
| | Serum | 75 | 1·90 to 7·99 | 1·00 | 0·04 | 0·11 |
| | Serum | 75 | 3·4 to 6·51 | 1·04 | −0·2 | 0·39 |
| CL (mmol/l) | Plasma | 90 | 93 to 114 | 1·03 | −2·63 | 2·20 |
| | Plasma | 90 | 93 to 112 | 1·12 | −10·10 | 2·44 |
| | Serum | 75 | 80 to 119 | 1·01 | −0·63 | 1·88 |
| | Serum | 74 | 84 to 112 | 1·13 | −10·71 | 6·17 |
| IGA (g/l) | Serum | 100 | 1·00 to 2·46 | 1·00 | 0·03 | 0·03 |
| | Serum | 99 | 0·42 to 64·32 | 1·00 | 0·00 | 0·55 |
| FT (μg/l) | Plasma | 100 | 6 to 518 | 0·93 | 0·33 | 25·9 |
| TF (g/l) | Plasma | 90 | 1·00 to 6·00 | 0·95 | 0·14 | 0·28 |
| PHEBA (μg/ml) | Plasma | 70 | 1·2 to 59·6 | 1·00 | −0·60 | 1·63 |
| | Serum | 98 | 1·0 to 43·3 | 0·95 | −0·96 | 1·92 |
| | Serum | 80 | 12·4 to 45·2 | 1·08 | −2·44 | 2·42 |
| PHENY (μg/ml) | Plasma | 70 | 1·4 to 28·9 | 1·01 | −0·59 | 1·46 |
| | Serum | 99 | 0·3 to 60·2 | 1·00 | −0·71 | 1·72 |
| | Serum | 79 | 3·5 to 24·4 | 1·03 | −1·49 | 1·55 |
| THEO (μg/ml) | Plasma | 70 | 2·0 to 62·0 | 1·06 | −0·18 | 0·98 |
| | Serum | 100 | 0·3 to 26·7 | 1·08 | −0·21 | 0·77 |
| | Serum | 50 | 2·1 to 23·4 | 1·04 | −0·52 | 1·59 |
| DIG (ng/ml) | Serum | 98 | 0·0 to 4·4 | 0·75 | −0·06 | 0·76 |
| | Plasma | 100 | 0·3 to 3·5 | 1·03 | −0·14 | 0·18 |
| | Serum | 100 | 0·1 to 4·3 | 0·96 | 0·03 | 0·35 |
| | Serum | 80 | 0·4 to 4·4 | 1·03 | −0·09 | 0·40 |
| BNAG (U/l) | Urine | 100 | 0·5 to 7·5 | 1·01 | 0·07 | 0·10 |
| CREA (mmol/l) | Urine | 100 | 2·3 to 29·2 | 1·00 | −0·15 | 0·22 |
| | Urine | 100 | 1·5 to 18·8 | 1·00 | −0·20 | 0·50 |
| MAU (mg/l) | Urine | 100 | 1·5 to 1365 | 1·01 | 0·26 | 1·05 |
| | Urine | 90 | 0·0 to 67 | 0·73 | 1·49 | 3·09 |
| | Urine | 94 | 2·0 to 58 | 1·60 | −2·46 | 2·65 |
| NA (mmol/l) | Urine | 100 | 16 to 314 | 1·00 | 0·39 | 2·5 |
| | Urine | 100 | 9 to 230 | 0·98 | −1·35 | 4·1 |
| K (mmol/l) | Urine | 100 | 8 to 94 | 0·99 | 0·05 | 1·3 |
| | Urine | 100 | 8 to 92 | 0·99 | 0·24 | 2·2 |
| CL (mmol/l) | Urine | 100 | 12 to 286 | 1·01 | −2·03 | 2·6 |
| | Urine | 100 | 5 to 246 | 1·00 | −5·90 | 11·5 |

